# Veterinary Medical Association of New York County

**Published:** 1897-02

**Authors:** Robert W. Ellis

**Affiliations:** Secretary


### SOCIETY PROCEEDINGS.

### VETERINARY MEDICAL ASSOCIATION OF NEW YORK COUNTY.

THE regular monthly meeting was called to order December 2, 1896, at 8.45 P.M., at the Academy of Medicine, with the President, Dr. Huidekoper, in the chair. On roll-call the following members responded: Drs. Amling, C. C. Cattanach, J. J. Cattanach, J. S. Cattanach, J. S. Cattanach, Jr., Delaney, Ellis, Farley, Giffen, Gill, Huidekoper, Hanson, Loomes, Machan, MacKellar, Neher, O'Shea, and Ryder (18). The minutes of previous meeting were read and approved.

The Board of Censors (Dr. Gill, Chairman) reported favorably on the names of applicants for membership in the Association of the following gentlemen: Dr. Roscoe R. Bell, of Brooklyn, and Dr. F. E. Winslow, of Flushing, N. Y. The name of Dr. Frank H. Miller, of New York, was referred to the meeting as a worthy applicant, but not registered in the county. Moved and seconded that the report of the Board of Censors be accepted; carried. On vote, all three gentlemen were made members of the Association.

The Judiciary Committee (Dr. O'Shea, Chairman) reported that through our counsel, Messrs. Van Schaick and Norton, a prosecution had been made in the case of one N. S. Bryant, a quack from Kansas, who had issued circulars advertising himself as a "doctor" and "veterinary dentist," "specialist," and had been doing work in various parts of New York. After the arrest of said Bryant the case was heard by Judge Cornell at the Yorkville Court, and, after postponement, at the Harlem Court, Bryant was put under three hundred dollars bond, and the case was finally disposed of in special sessions, with conviction. Moved and seconded that the report be accepted; carried.

The Chair reported that the lists of the registered veterinarians of New York and Kings County had been obtained, and suggested that as the publication of this list would amount to a considerable expense it might be advisable to extend the publication still further by adding other information. (See prospectus, pages 11 to 14, JOURNAL advertising pages, for January, 1897.) The Chair believed that subscriptions and advertisements to this publication would at least pay for it, and would probably bring in a profit. Moved and seconded that the report be accepted and the matter placed in the hands of a committee of three for publication; carried.

Election of officers for the ensuing year: Nomination for President: Nomination of Dr. Huidekoper. Moved and seconded that the nominations close; carried. For Vice-President: Nominations of Drs. Robertson and Ryder. Moved and seconded that the nominations close; carried. For Secretary: Nomination of Dr. Ellis. Moved and seconded that the nominations close; carried. For Treasurer: Nomination of Dr. C. C. Cattanach. Moved and seconded that the nominations close; carried. As there was but one nomination for President, Secretary, and Treasurer, it was moved and seconded that the by-laws be suspended and vote by acclamation for these officers; carried. Ballot was next cast for Vice-President as follows: Dr. Robertson received thirteen and Dr. Ryder five votes. Moved and seconded that the election be declared unanimous for Dr. Robertson; carried.

According to a motion passed at the November meeting a list of all members eighteen months in arrears was taken up for action and disposed of in accordance with Article XII. of the by-laws.

A communication was then read from an expelled member of the Association and referred to the Board of Censors to report on at the next meeting.

The President appointed the following committees for the ensuing year, the officers being *ex-officio* members of each. Board of Censors: H. D. Gill, Chairman; J. E. Ryder, H. D. Hanson, J. S. Cattanach, R. R. Bell. Judiciary Committee: Arthur O'Shea, Chairman; Hanson, Jackson. Publication Committee: H. D. Gill, Chairman; J. E. Ryder, T. Delaney. Moved and seconded that the meeting adjourn; carried.

The regular monthly meeting was called to order at 8.45 P.M., January 6, 1897, at the Academy of Medicine, with the President, Dr. Huidekoper, in the chair. The following members responded to roll-call: Drs. Amling, Bell, C. C. Cattanach, J. S. Cattanach, J. S. Cattanach, Jr., Caulfield, Ellis, Farley, Foy, Gill, Huidekoper, Hanson, Jackson, Loomes, Lellman, Neher, O'Shea, Robertson, Ryder, Sherwood, and Winslow (21). The minutes of the previous meeting were read and approved.

The Board of Censors (Dr. Gill, chairman) reported that as the question of the legality of the expulsion of H. Clay Glover from the Association had been referred back to the Board for their consideration, they wished to submit the following suggestions to the meeting, to wit, that the secretary be authorized to send H. Clay Glover a complete copy of the charges and request him to appear before the Board at the next meeting to answer the same, said notification to be sent by registered letter. Moved and seconded that the report be accepted; carried.

Dr. Lellman then reported a case of "Polyneuritis of a Dog". Moved by Dr. O'Shea and seconded by Dr. Robertson that a vote of thanks be tendered to Dr. Lellman for his report. Dr. Huidekoper then gave a most interesting discourse on "Strains". A discussion followed, which was participated in by Drs. Neher, Robertson, Farley, Gill, and others. Moved and seconded that a vote of thanks be tendered Dr. Huidekoper; carried.

Dr. O'Shea (chairman Judiciary Committee) read the following communication from the Association's Counsel, addressed to the President:

NEW YORK, December 19, 1896.

"We beg to thank you for your letter of the 15th, enclosing check. We have sent a receipted bill to Dr. Cattanach, as requested. We beg to enclose an affidavit in the Bryant case, which Magistrate Cornell said could not be improved upon. We can no doubt adapt it to fill such other cases as may arise. In this connection we may call your attention to the fact that we have serious doubts whether, under the provisions of the Act of 1895, a person could be held as for the commission of a misdemeanor for merely operating without registration. Magistrate Cornell intimated that this was his view, and that he would probably have to discharge Bryant if it had not been proved that Bryant appended a veterinary title to his name.

"It would seem that the only remedy that the Act provides for the practising without registration was a civil suit for the recovery of a money-penalty for such offence. In view of this we can suggest that it might be advisable, in any case where you could prove that the person complained of has assumed a veterinary title, that you should consult with us before proceeding to arrest him.

"We are examining the papers in the Benson case, and will send you our conclusion in a few days. The question involved is an important one, and is not free from difficulties. With reference to the Mulvey case, we have no doubt that the registration would be cancelled on proper application being made. Do you wish us to proceed?

"Very truly yours,

"VAN SCHAICK & NORTON."

(AFFIDAVIT.)

"POLICE COURT, CITY OF NEW YORK.:

"The People of the State of New York on the Relation of 'The Veterinary Medical Association of New York County,' *plaintiff*, against Newton S. Bryant, the name Newton being fictitious, defendant's real name being unknown, *defendant*.

"CITY AND COUNTY OF NEW YORK, *ss.*:

"Rush S. Huidekoper, being duly sworn, doth depose and say that he is the President of the New York Veterinary Medical Association of New York County; that he hereby makes a complaint against the above-named Newton S. Bryant, the name Newton being fictitious, said Bryant's first name being unknown to the deponent, and charges said Bryant with the following offences against the laws and statutes of the State of New York:

"*First.* That the said Bryant is practising veterinary medicine and surgery within the county of New York without being previously registered and legally authorized or licensed by the Regents and registered as required by the provisions of chapter 860, sections 171 and 181, of the Laws of 1895 as amended by chapter 840 of the Laws of 1896; that said Bryant has practised as follows: On or about October 24, 1896, deponent is informed and believes he operated on five horses in the stable of one John Doyle, of 140 West 54th street; that on or about October 10, 1896, he operated on the horses of one Protor, at Tattersalls, at 55th street and 7th avenue; that in or about the last week of September, 1896, he operated on the horses belonging to one Frank Work, at his stables, 157 West 56th street; that on or about October 10, 1896, he operated on a number of horses at the stable of Strauss & Hexter, at 1722 Broadway; that on or about October 19 or 20, 1896, he operated on horses at Tattersalls, at 55th street and 7th avenue.

"That said statutes provide, in chapter 860 of the Laws of 1895, section 184, that any person guilty of violating any of the other provisions of this Act not otherwise specifically punished herein . . . shall be guilty of a misdemeanor.

"*Second.* That said Bryant, without having been authorized to do so legally, appends a veterinary title to his name, and assumes and advertises a veterinary title in such manner as to convey the impression that he is a lawful practitioner of veterinary medicine or of one of its branches; that said Bryant issues and advertises his business by means of a printed circular which deponent has seen and which said Bryant has delivered to various persons known to deponent, upon the first page of which is the following inscription: 'Dr. N. S. Bryant, veterinary dentist'.

"That chapter 860 of the Laws of 1895, in section 184, provides, 'and any person who shall, without having been authorized to do so legally, append any veterinary title to his or her name, or shall assume or advertise any veterinary title in such a manner as to convey the impression that he is a lawful practitioner of veterinary medicine or any of its branches, shall be guilty of a misdemeanor.'"

The Committee also assured the members of the Association that a bill would be introduced by the committee this winter for the exemption from jury-duty of veterinary practitioners of New York and Queens Counties. Moved and seconded that the report be accepted; carried.

President Huidekoper reported on the progress of the *Blue-book.*

An application for honorary membership was submitted as follows: We hereby propose Prof. Alexander Liautard for honorary membership in the Veterinary Medical Association of New York County, as his life-work has been devoted entirely to the elevation and advancement of the veterinary profession in this county. Signed by Arthur O'Shea, J. L. Robertson, H. D. Hanson. This was referred by the chair to the Board of Censors.

The secretary next read the resignation from the Association of Dr. Thomas Giffin; moved and seconded that the report be accepted. Division of vote; moved and seconded that the matter be laid over until the next meeting; carried.

Moved and seconded that the meeting adjourn; carried.

ROBERT W. ELLIS, D.V.S.,
Secretary.

